# Supplementary material for: Genomic Loci for Sclerotinia Stem Rot Resistance and Chlorophyll Stability in Brassica napus: Integrating GWAS With Microbiome Insights
Source: Plant Environ Interact. 2025 Oct 24;6(5):e70092. doi: 10.1002/pei3.70092 (PMC12550765; doi:10.1002/pei3.70092)
Supplement: Supplementary file 1 — Figure S1: Sclerotinia stem rot lesion length (LL), lesion area (LA) and relative lesion area (RLA) growth (A), and chlorophyll content before Sclerotinia infection (SPADH), after infection (SPADI) and chlorophyll index (CI) values (B) in the most resistant and susceptible accessions of oil seed rape. [file PEI3-6-e70092-s003.pdf]

Supplementary Figures

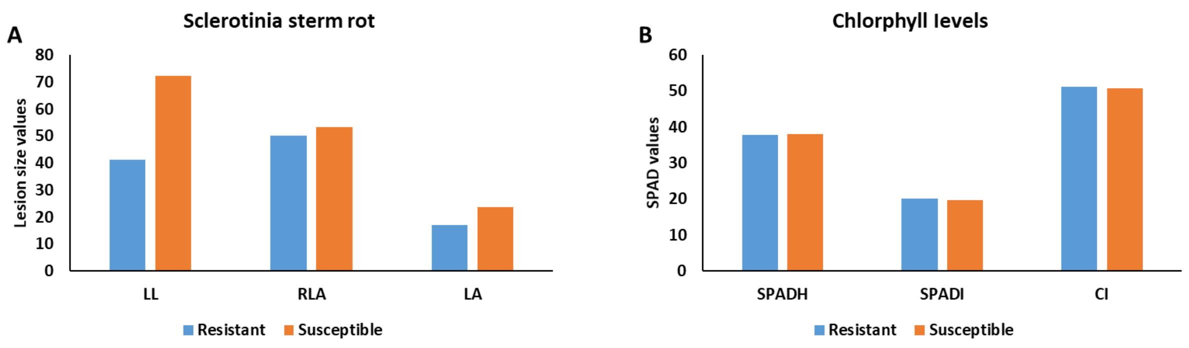

Supplementary Figure 1. Sclerotinia stem rot lesion length (LL), Lesion area (LA) and Relative lesion area (RLA) growth (**A**), and chlorophyll content before Sclerotinia infection (SPADH), after infection (SPADI) and Chlorophyll Index (CI) values (**B**) in the most resistant and susceptible accessions of oil seed rape.
